# Systematic review of clinical practice guidelines for colorectal and anal cancer: the extent of recommendations for managing long-term symptoms and functional impairments

**DOI:** 10.1007/s00520-020-05301-7

**Published:** 2020-02-05

**Authors:** Lisette M. Wiltink, K. White, M. T. King, C. Rutherford

**Affiliations:** 1 School of Psychology, Quality of Life Office, Faculty of Science, The University of Sydney, Level 6 North, Chris O’Brien Lifehouse C39Z, Sydney, NSW 2006 Australia; 2 Susan Wakil School of Nursing and Midwifery, Cancer Nursing Research Unit (CNRU), Faculty of Medicine and Health, The University of Sydney, Sydney, Australia; 3 Department of Radiation Oncology, Leiden University Medical Center, Leiden, Netherlands

**Keywords:** Clinical practice guidelines, Anal cancer, Colorectal cancer, Long-term side effects, Long-term symptoms, Long-term functioning

## Abstract

**Purpose:**

Due to increasing numbers of colorectal and anal cancer survivors, more individuals are living with long-term symptoms after treatment. A systematic review was undertaken to assess the extent to which practice guidelines for colorectal and anal cancer provide recommendations for managing long-term symptoms and functioning impairments.

**Methods:**

Four electronic databases and websites of 30 international cancer societies were searched for clinical practice guidelines, consensus statements, or best practice recommendations for colorectal or anal cancer. Quality of included guidelines was evaluated with the Appraisal of Guidelines for Research & Evaluation II tool. Results were narratively summarized.

**Results:**

We included 51 guidelines or consensus statements. Recommendations for managing long-term symptoms or functioning impairments were reported in 13 guidelines (25.4%). All 13 recommend a healthy lifestyle, diet, body weight, and physical activity. The ASCO Colorectal Cancer Survivorship Care Guideline is the most comprehensive, including interventions targeting sexual and bowel function to pain and cognitive issues, and also highlights limited evidence for informing management strategies. Other guidelines recommend treating incontinence, chronic diarrhea, and distress, and stress the need for greater awareness for sexual dysfunction, survivorship clinics, and referrals to specific supportive care interventions.

**Conclusions:**

Few clinical practice guidelines include recommendations for managing long-term symptoms and functioning impairments. It is unclear if this is due to limited evidence or absence of management strategies and interventions. Clear recommendations for managing long-term symptoms and functioning to help health professionals in supporting colorectal and anal cancer survivors are needed.

## Introduction

Colon and rectal cancer combined are the third most commonly diagnosed cancer worldwide [1]. In 2018, 1,849,518 new cases of colon or rectal cancer were diagnosed, with an additional 48,541 anal cancers diagnosed [2]. The highest incidence rates are in developed countries, such as Australia with 16,171 newly diagnosed colorectal cancer patients and 470 anal cancer patients in 2018 [3]. Improvements in screening and treatment for bowel, colon, and anal cancer, referred to hereafter as colorectal cancer (CRC), have contributed to a growing population of cancer survivors with unmet needs, long-term symptoms, and functioning impairments after treatment that can negatively impact on health-related quality of life (HRQL) [4, 5].

Multiple long-term symptoms can be experienced after treatment for CRC. For bowel symptoms, these include rectal bleeding, diarrhea, fecal frequency, incontinence and urgency, and rectal tenesmus (feeling of incomplete defecation) [6]. Other common symptoms are chemotherapy-induced neuropathy, insomnia, fatigue, cognitive dysfunction, issues with body image, psychological distress, [7, 8], and sexual dysfunction [5]. All long-term symptoms contribute to increased symptom burden, in terms of both severity and frequency, which in turn is associated with decreased HRQL [9]. In order to decrease symptom burden and improve HRQL, evidence-based interventions should be used to manage long-term symptoms and functioning impairments. Since clinicians are often unaware of interventions designed to detect and manage symptoms [6], this systematic review aimed to investigate the extent to which clinical practice guidelines for CRC provide recommendations for managing long-term symptoms and functioning impairments following treatment for CRC.

## Methods

Our systematic review was conducted according to the Preferred Reporting Items for Systematic Reviews and Meta-Analyses (PRISMA) guidance [10]. The following 4 electronic databases were searched using the OVID web gateway: MEDLINE, EMbase, PsycINFO, and CINAHL from inception to 26th of July 2019. The search strategy included terms for colorectal cancer and clinical practice guidelines. Details of the search strategy are reported in Appendix 1. To supplement this, reference lists of studies identified were used to find other relevant guidelines, and 33 international cancer societies and guideline organization websites were searched for additional guidelines, consensus statements, or best practice recommendations.

### Eligibility and inclusion criteria

Eligible guidelines were CRC-specific, written in English, and contained information about how to manage or treat long-term symptoms and functioning impairments after treatment. In cases where there was a chronological sequence of versions of a guideline released by a particular organization, only the most recent version of the guideline was reviewed.

### Quality assessment

The Appraisal of Guidelines for Research & Evaluation (AGREE) II tool was used to assess the quality of eligible guidelines, i.e., containing information about how to manage or treat long-term symptoms and functioning impairments after treatment [11]. The AGREE II tool consists of 6 domains with 21 quality and two global rating items with 7 point-categories: 1 = did not meet criteria to 7 = met criteria. Quality items relating to guideline rigor, competing interests, and implementation were of particular relevance to our aims as these domains relate to the identification of the best possible evidence to inform the guidelines.

### Data extraction and analysis

Data pertaining to country, year of publication, organization, title, and recommendations for management strategies or interventions for long-term symptoms and functioning impairments were extracted. A narrative synthesis of recommendations was conducted.

## Results

**Fig. 1:**
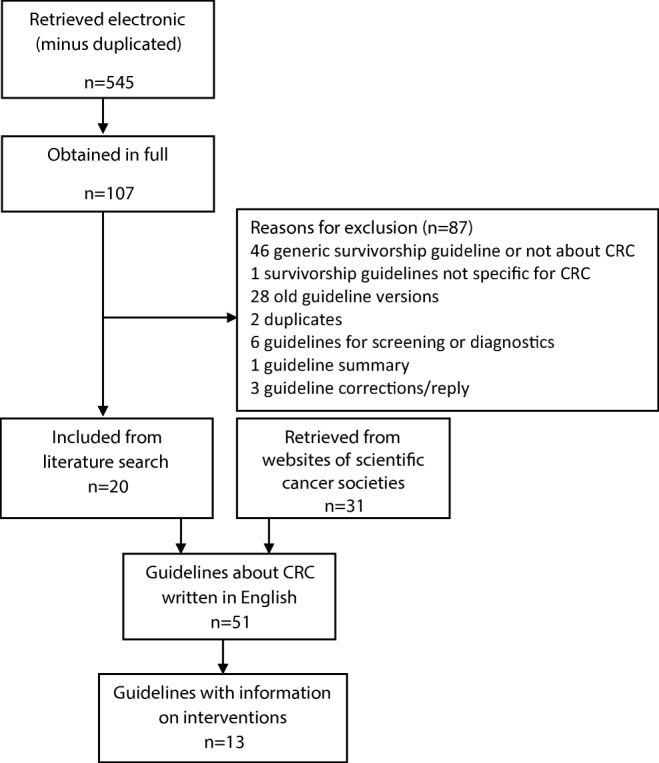
Flowchart of included guidelines

The searches retrieved 545 papers of which 20 were potentially eligible for inclusion. An additional 31 publications were retrieved through searches of international websites (Fig. 1). Fifty-one guidelines for CRC were identified (Appendix Table 3). Major international organizations that published these include the National Comprehensive Cancer Network (NCCN), American Society for Clinical Oncology (ASCO), and the European guidelines of the European Society for Medical Oncology (ESMO), European Society of Surgical Oncology (ESSO), and European Society of Radiotherapy and Oncology (ESTRO). We found nine guidelines specific to colon cancer, nine for rectal cancer, and five for anal cancer, and twenty-eight guidelines providing recommendations for both colon and rectal cancer.

If information on surveillance was included in the guidelines, it emphasized recurrence detection during follow-up. Only 13 of the 51 guidelines (25.4%) met the eligibility criterion of containing information about how to manage or treat long-term symptoms and functioning impairments after treatment (Table 1).

**Table 1:** Colorectal and anal cancer guidelines that include information on interventions to manage or treat long-term symptoms and functioning impairments after treatment

Country, year first published	Organization	Title	Intended population	Type
Australia, 2017	NHMRC	Clinical practice guidelines for the prevention, early detection, and management of colorectal cancer	Colorectal cancer	CPG
Canada,2019	Alberta Provincial GI Tumour Team	Colorectal Cancer Surveillance (Stages I, II, and III)	Colorectal cancer	CPG
Europe, 2013	ESMO	Early colon cancer: ESMO clinical practice guidelines for diagnosis, treatment and follow-up	Colon cancer	CPG
Europe, 2012	ESMO	ESMO consensus guidelines for management of patients with colon and rectal cancer. A personalized approach to clinical decision making	Colorectal cancer	consensus guideline
Europe, 2014	ESMO, ESSO, ESTRO	Anal cancer: ESMO-ESSO-ESTRO Clinical practice guidelines for diagnosis, treatment and follow-up	Anal cancer	CPG
Europe, 2017	ESMO	Rectal cancer: ESMO Clinical Practice Guidelines for diagnosis, treatment and follow-up	Rectal cancer	CPG
Germany, 2019	German Guideline Program in Oncology	Evidenced-Based Guideline for Colorectal Cancer	Colorectal cancer	CPG
Canada, 2012	Cancer Care Ontario	Follow-up care, surveillance protocol, and secondary prevention measures for survivors of colorectal cancer	Colorectal cancer	CPG
USA, 2015	American Cancer Society	American Cancer Society Colorectal Cancer Survivorship Care Guidelines	Colorectal cancer	CPG
USA, 2018	NCCN	Anal Carcinoma, Version 1.2019	Anal cancer	CPG
USA, 2018	NCCN	Colon cancer (MENA edition) v 4.2018	Colon cancer	CPG
USA, 2019	NCCN	Rectal cancer, version 2.2019	Rectal cancer	CPG
USA, 2019	NCCN	Colon cancer, version 2.2019	Colon cancer	CPG

*GI*, gastrointestinal; *CPG*, clinical practice guideline; *ESMO*, European Society for Medical Oncology; *ESSO*, European Society of Surgical Oncology; *ESTRO*, European Society of Radiotherapy and Oncology; *NHMRC*, National Health and Medical Research Council; *NCCN*, National Comprehensive Cancer Network

### Quality assessment

**Fig. 2:**
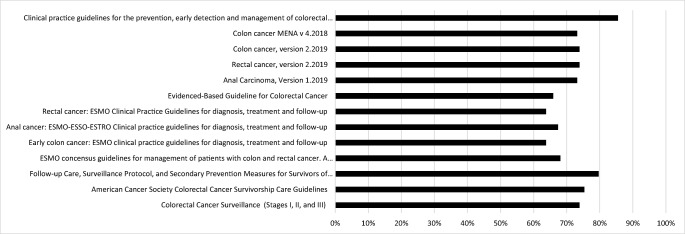
Quality assessment score (%) according to AGREE II for 13 guidelines that included information on interventions to manage or treat long-term symptoms and functioning impairments after treatment. ESMO, European Society for Medical Oncology; NHMRC, National Health and Medical Research Council; NCCN, National Comprehensive Cancer Network; GI, gastrointestinal

**Fig. 3:**
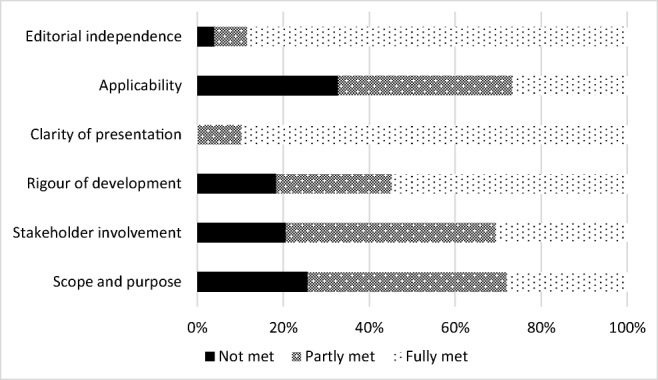
Proportion of 13 guidelines with information on interventions meeting each AGREE II quality criterion

The overall quality of the 13 eligible guidelines was high according to the AGREE II criteria (Fig. 2); however, none of the guidelines met all quality criteria. The lowest quality scores related to applicability (e.g., usefulness of guidelines for daily practice), likely barriers/facilitators to implementation, and strategies to improve uptake (Fig. 3).

### Information on interventions for long-term symptoms

The American Cancer Society Colorectal Cancer Survivorship Care Guideline is the most extensive, providing detailed information about several issues (Table 2). Long-term symptoms are described and possible interventions outlined. However, the guideline acknowledges that evidence is limited to support treatment, and sometimes management of long-term symptoms/function is based on expert opinion. Moreover, a healthy weight, physical activity, and a multi-fiber diet with low amounts of saturated fat are encouraged [12]. The Alberta Colorectal Cancer Surveillance (stages I, II, and III) guideline refers to the ASCO colorectal cancer survivorship care guideline for information about long-term symptom/functioning management and includes healthy lifestyle promotion recommendations [13].

**Table 2:** Topics covered in the recommendations for managing long-term symptoms and functioning impairments of the American Cancer Society Colorectal Cancer Survivorship Care Guideline

Gastrointestinal	
Cardiovascular	
Cognitive	
Dental and oral	
Distress, depression and anxiety	
Fatigue	
Neuropathy	
Ostomy and stoma	
Pain	
Sexual function and fertility	
Urinary and bladder	

Four guidelines are published by the NCCN. The survivorship section of the guidelines for rectal cancer, colon cancer, and the Middle East and North Africa (MENA) version for colon cancer emphasize the importance of defined roles of care givers in the surveillance period to detect local recurrences in time and state “for chronic diarrhea or incontinence: consider anti-diarrheal agents, bulk-forming agents, diet manipulation, pelvic floor rehabilitation, and protective undergarments” [14–17]. Moreover, involvement in ostomy support groups or coordination by an ostomy care specialist can be considered, and undergoing national health-screenings, a healthy body weight, physical activity, healthy diet, and not smoking are recommended. Daily aspirin of 325 mg could be considered for secondary prevention. These four guidelines refer readers to NCCN survivorship guidelines for distress, pain, neuropathy, fatigue, sexual dysfunction, or precautions involving physical activity, and to the NCCN guidelines for Distress Management for issues around body changes following treatment for cancer. Further, several guidelines recommend monitoring of long-term symptoms, and functioning impairments should be organized by the primary care physician, who are oncologists in the MENA region [14–17]. Additionally, in the rectal and anal cancer guidelines, screening for sexual dysfunction and urinary dysfunction is recommended, and referral to a urologist or gynecologist should be made if symptoms persist. Also, bone density monitoring should be considered due to the potential for pelvic fractures after radiotherapy [15, 16]. For oxaliplatin-induced neuropathy, duloxetine is advised or therapies such as heat, ice, or acupuncture [14, 16, 17].

Four CRC guidelines with information on survivorship were published by ESMO. In these guidelines, the use of late effects/survivorship clinics after pelvic radiation is recommended [2, 18–20]. For anal cancer, patient-reported outcome data is scarce, but suggests attention to sexual dysfunction is needed. Pelvic floor exercises and biofeedback training are recommended to treat fecal urgency and incontinence [2]. After treatment for rectal cancer, lower genitourinary toxicity and social, financial, and emotional aspects should be addressed to maximize well-being, but recommendations for how to address these issues are not provided [18]. Only the guideline for early colon cancer describes how to treat cancer sequelae and highlights that assessment of medical and psychological late effects are a major part of survivorship care. Dietary counseling and use of over-the-counter medications such as fiber, laxative, stool softeners, and antidiarrheals are recommended. Employment, financial concerns, and distress should also be assessed [19]. Further, all four guidelines emphasize a healthy lifestyle to prevent co-morbidity [2, 18–20].

The Cancer Care Ontario Follow-up care guideline for survivors of colorectal cancer states that despite a lack of high-quality evidence for secondary prevention, patients should be counseled to have a healthy lifestyle, body weight and diet, and to participate in physical activity [21]. This is also recommended in the German guideline with the addition that “patients benefit if they can take the management of their symptoms and side effects into their own hands” [22]. However, recommendations for how to self-manage symptoms are not provided. A separate section on psychological care and treatments is included in the Australian guideline of the National Health and Medical Research Council (NHMRC). It recommends routine screening with the Distress Thermometer and the Edmonton Symptom Assessment Schedule and provides a range of evidence-based psychological interventions if required. These include relaxation-based, cognitive behavioral, and supportive-expressive therapies. Early referral to a psychologist or liaison psychiatrist is advised, and also peer support was accepted and appreciated by CRC survivors. [23]

## Discussion

Of the 51 CRC-specific guidelines identified, only 13 (25%) contained recommendations for how to manage some long-term symptoms and functioning impairments following treatment for CRC. These 13 guidelines were published by the NCCN, ESMO, ASCO, Cancer Care Ontario, Alberta provincial GI tumor team, German Guideline program in oncology, and the NHMRC. All 13 guidelines recommend a healthy lifestyle, diet and body weight, and physical activity. The ASCO guideline provides the most comprehensive coverage of interventions ranging from cognitive and pain to bowel and sexual issues, and also acknowledges that evidence is limited to support base treatment guidelines. Other guidelines include some suggestions for treating chronic diarrhea, incontinence and psychological distress, and highlight need for greater awareness for sexual dysfunction, survivorship clinics, and referrals to specific supportive care interventions. Thus, few clinical practice guidelines for CRC provide a comprehensive range of recommendations for how to manage or treat long-term symptoms and functioning impairments following treatment for CRC*.*

The main focus of survivorship care in the guidelines is on detection of early recurrence, the timing of follow-up, and which diagnostic tools to use. Much less attention is paid to long-term symptoms and functioning impairment, or to the interventions recommended to manage and alleviate these issues to reduce patients’ symptom burden and improve HRQL. This is a major gap that needs to be filled as previous research demonstrates a link between HRQL and survival. In a large study of 1074 CRC patients, depressive symptoms significantly increased mortality risk in the first 2 years after diagnosis (hazard rate, 2.55, *p* = 0.001) with a hazard rate of 1.88 (*p* < 0.01) up to 10 years after diagnosis [24]. During the last few decades, more attention is being paid to developing interventions for late symptoms after cancer treatment. For example, Andreyev et al. reported that a gastroenterologist- or nurse-led algorithm-based treatment resulted in a decrease of bowel symptoms compared with a self-help booklet [25]. Also, interventions are emerging for sexual dysfunction after pelvic radiotherapy, such as mindfulness and cognitive behavioral therapy, couple therapy, scheduled intimacy, vaginal dilator therapy and moisturizers, hormone replacement, and phosphodiesterase type 5 inhibitors [26]. Research is needed to further assess interventions for long-term symptoms and functioning impairments. Moreover, guidelines recommend screening for distress and symptoms, but do not state if patient-reported outcomes could or should be used for routine screening. A number of trials have shown benefit of using patient-reported outcomes for routinely screening during treatment [27, 28]; their potential benefit in long-term follow-up of CRC should be investigated.

Several guidelines refer to generic survivorship guidelines. While these were excluded from our review, we note that the 2019 NCCN survivorship guideline contains information about long-term symptoms and treatment of cardiotoxicity, depression, anxiety, cognitive function, fatigue, lymphoedema, hormone problems, pain, sexual function, and sleep, independent of the location of the primary cancer. Unfortunately, interventions for gastrointestinal problems are not included [29]. NCCN regularly updates all guidelines. The NCCN guidelines for colon and rectal cancer met our inclusion criteria regarding information on long-term adverse effects after treatment and possible interventions, but in an older version (the NCCN CRC guideline), such information was not provided. The inclusion of this information in the updated guideline suggests rising international awareness for late effects and HRQL.

This systematic review highlights the limited guidance available on how to manage long-term symptoms and functioning impairments in CRC survivors. This knowledge gap has implications for both clinicians, patients, and their informal caregivers. A previous review showed that many CRC survivors find ways to self-manage their symptoms through trial and error rather than seek professional help [30]. Self-help strategies are often not evidence-based, so it may not be effective and perhaps even detrimental, worsening symptoms or impairment. It is not clear why survivors choose to self-manage, but the absence of recommendations for clinicians in clinical practice guidelines may at least in part be a contributing factor. Moreover, none of the guidelines addressed the need to support informal caregivers in managing symptoms. As mentioned above, interventions have been developed to manage several symptoms and functions caused by treatment for cancer, yet expert opinions are often used to inform clinical practice guidelines rather than evidence from clinical effectiveness trials. It is unclear whether the use of expert opinion is due to limited effectiveness evidence. Future research should focus on synthesizing all available evidence on supportive care interventions and management strategies, and guidelines need to be updated periodically to incorporate emerging evidence. Another recommendation, also stated in a few guidelines, is the use of survivorship clinics. Literature shows that patients are highly satisfied with the services of such a clinic, mainly due to the multidisciplinary approach, the time taken to address the symptoms, and the referrals to appropriate supporting programs [31].

Strengths of this review are the inclusion of international guidelines, a thorough search of both electronic databases and websites for 33 cancer societies, and guideline quality assessment against the criteria of the AGREE II tool. Two limitations of our review were the inclusion of only guidelines available in English, and with our search process, we could not accurately count the number of non-English guidelines excluded. Some non-English guidelines that we did not have resources to review may provide recommendations for long-term symptoms or functions. For example, the Dutch national guideline for CRC (which we are aware of as LW is a Dutch clinician) provides risk factors for fecal incontinence, recommends the use of diagnostic tools to evaluate long-term symptoms, and provides interventions for managing specific problems such as fecal incontinence with bulk-forming agents or loperamide to decrease fecal frequency [32]. However, despite excluding non-English guidelines, we expect that they would be informed by the same international evidence that informed guidelines published in English.

In conclusion, the majority of guidelines for CRC reviewed did not provide recommendations for management of long-term symptoms and functioning impairment following treatment for CRC. Their recommendations primarily focus on detection of early recurrence, the timing of follow-up, which diagnostic tools to use, and on the importance of a healthy diet and weight, and active lifestyle after treatment. Few recommendations are available for how to reduce or manage symptom burden and improve HRQL. This may be due to lack of evidence about effectiveness of potential management strategies. There is need for clear evidence-based recommendations for managing long-term symptoms and functioning impairments to assist health professionals in supporting CRC survivors and to ameliorate suffering due to persistent symptoms and functioning impairments that often go unmanaged.

## Appendix 1. Systematic search strategy

### Topic (colorectal cancer)

1. (colorectal adj1 (cancer* or neoplasm* or tumo$r$ or carcinoma$)).ti,ab,mp
2. (bowel adj1 (cancer$ or neoplasm$* or tumo$r$ or carcinoma$)).ti,ab,mp
3. (colon adj1 (cancer$ or neoplasm$ or tumo$r$ or carcinoma$)).ti,ab,mp
4. (anal adj1 (cancer$ or neoplasm$ or tumo$r$ or carcinoma$)).ti,ab,mp
5. ((rectal or rectum or anal) adj1 (cancer$ or neoplasm$ or tumo$r$ or carcinoma$)).ti,ab,mp
6. or/1-6

### Outcomes

7. (clinical practice guideline*).ti,ab,kw
8. (clinical guideline*).ti,ab
9. (best practice guideline*).ti,ab
10. ((survivorship or supportive care) guideline*).ti,ab,kw
11. (treatment recommendation*).ti,ab
12. (evidence$based recommendation*).ti,ab
13. (clinical pathway).ti,ab
14. (care pathway*).ti,ab,kw.
15. or/7-14

### Exclusion

16. Prevention
17. (p$ediatric* or child* or infant* or youth* or adolescen*).ti,ab.
18. animal*.mp,ti,ab.
19. (mouse or rat or mice).ab.
20. (in vivo or in vitro).mp.
21. ((semi-structur* or semistructur* or unstructur* or informal or in-depth or indepth or face-to-face or structur* or guide) adj3 (interview* or discussion* or questionnaire*)).ti,ab.
22. interviews as topic/
23. (focus group* or qualitative or ethnograph* or fieldwork or “field work” or “key informant”).ti,ab.
24. focus groups/ or narration/ or qualitative research/
25. (retraction of publication or retracted publication).pt.
26. (cohort analysis or cohort study).pt,ab,ti,kw.
27. longitudinal stud*.pt,ab,ti,kw.
28. retrospective.ti,ab.
29. Epidemiolog*.ti,ab.
30. cross-section*.ti,ab.
31. Case Control Study/ or Control Group/
32. (case$ and control$).mp,tw.
33. (case$ and series).tw.
34. Matched-Pair Analysis/
35. (animal* not human*).sh.
36. historical article.pt.
37. review.pt.
38. letter.pt.
39. comment.pt.
40. editorial.pt.
41. or/16-40
42. 6 and 15
43. 42 not 41

## Appendix 2

**Table 3:** All retrieved guidelines about colorectal and anal cancer written in English

Country, year first published	Organization	Title	Intended population
Australia, 2017	NHMRC	Clinical practice guidelines for the prevention, early detection and management of colorectal cancer	Colorectal cancer
Canada, 2012	Cancer Care Ontario	Follow-up care, surveillance protocol, and secondary prevention measures for survivors of colorectal cancer	Colorectal cancer
Canada, 2013	Alberta Provincial GI Tumor Team	Anal cancer	Anal cancer
Canada, 2013	Alberta Provincial GI Tumor Team	Early stage rectal cancer	Rectal cancer
Canada, 2014	Cancer Care Ontario	Strategies of Sequential Therapies in Unresectable, Metastatic Colorectal Cancer Treated with Palliative Intent	Colorectal cancer
Canada, 2015	Cancer Care Ontario	Continuous versus Intermittent Chemotherapy Strategies in Inoperable, Advanced Colorectal Cancer	Colorectal cancer
Canada, 2016	Cancer Care Ontario	Adjuvant systemic chemotherapy for stages II and III colon cancer after complete resection: a clinical practice guideline	Colon cancer
Canada, 2017	Alberta Provincial GI Tumor Team	Early stage colon cancer	Colon cancer
Canada, 2018	Alberta Provincial GI Tumor Team	Metastatic colorectal cancer	Colorectal cancer
Canada, 2018	Cancer Care Ontario	The Role of Primary Tumor Location in the Selection of Biologics for the Treatment of Unresectable Metastatic Colorectal Cancer: An Endorsement of a Canadian Consensus Statement	Colorectal cancer
Canada, 2019	Cancer Care Ontario	Preoperative or Postoperative Therapy for the Management of Patients With Stage II or III Rectal Cancer	Rectal cancer
Canada,2019	Alberta Provincial GI Tumor Team	Colorectal Cancer Surveillance (Stages I, II, and III)	Colorectal cancer
Europe 2013	ESMO	Early colon cancer: ESMO clinical practice guidelines for diagnosis, treatment and follow-up	Colon cancer
Europe, 2011	EAES	Laparoscopic extraperitoneal rectal cancer surgery: the clinical practice guidelines of the european association for endoscopic surgery	Rectal cancer
Europe, 2012	ESMO	ESMO concensus guidelines for management of patients with colon and rectal cancer. A personalized approach to clinical decision making	Colorectal cancer
Europe, 2014	ESMO, ESSO, ESTRO	Anal cancer: ESMO-ESSO-ESTRO Clinical practice guidelines for diagnosis, treatment and follow-up	Anal cancer
Europe, 2015	European Association for Endoscopic Surgery	Early Rectal Cancer: The European Association for Endoscopic Surgery (EAES) Clinical Consensus Conference	Rectal cancer
Europe, 2016	ESMO	ESMO concensus guidelines for management of patients with metastatic colorectal cancer	Colorectal cancer
Europe, 2017	ESMO	Rectal cancer: ESMO Clinical Practice Guidelines for diagnosis, treatment and follow-up	Rectal cancer
Europe, 2018	JSMO-ESMO	Pan-asian adapted ESMO consensus guidelines for the management of patients with metastatic colorectal cancer: a JSMO-ESMO initiative endorsed by CSCO, KACO, MOS, SSO and TOS	Metastatic colorectal cancer
France, 2011	SFCD, ACHBT	Management of patients with synchronous liver metastases of colorectal cancer. Clinical practice guidelines. Guidelines of the French society of gastrointestinal surgery (SFCD) and of the association of hepatobiliary surgery and liver transplantation (ACHBT). short version.	Metastatic colorectal cancer
France, 2017	SNFGE, FFCD, GERCOR, UNICANCER, SFCD, SFED, SFRO	Rectal cancer: French intergroup clinical practice guidelines for diagnosis, treatments and follow-up (SNFGE, FFCD, GERCOR, UNICANCER, SFCD, SFED, SFRO)	Rectal cancer
Germany, 2009	AWMF, DKH, DKG	Colorectal carcinoma: the management of polyps, (neo) adjuvant therapy, and the treatment of metastases	Colorectal cancer
Germany, 2019	German Guideline Program in Oncology	Evidenced-Based Guideline for Colorectal Cancer	Colorectal cancer
Greece 2016	HESMO	Clinical practice guidelines for the surgical management of colon cancer: a consensus statement of the hellenic and cypriot colorectal cancer study group by the HESMO	Colon cancer
Italy, 2007	Italian Society of Colorectal surgery	Laparoscopic surgery for colorectal cancer: clinical practical guidelines of the italian society of colorectal surgery	Colorectal cancer
Japan, 2015	Japan Gastroenterological Endoscopy Society	JGES Guidelines for Colorectal Endoscopic Submucosal Dissection/Endoscopic Mucosal Resection	Colorectal cancer
Japan, 2019	Japanese society for cancer of the colon and rectum	Japanese Society for Cancer of the Colon and Rectum (JSCCR) guidelines 2019 for the treatment of colorectal cancer	Colorectal cancer
New Zealand, 2011	New Zealand Guidelines Group	Management of early colorectal cancer	Colorectal cancer
Saudi Arabia, 2014	Saudi Oncology Society	Saudi Oncology Society clinical management guideline series Colorectal cancer 2014	Colorectal cancer
Singapore, 2015	Singapore Cancer Network	Singapore Cancer Network (SCAN) guidelines for systemic therapy of colorectal cancer	Colorectal cancer
Spain 2011	SEOM	SEOM clinical guidelines for the treatment of anal cancer	Anal cancer
Spain 2013	SEOM	SEOM clinical guidelines for the adjuvant treatment of colorectal cancer 2013	Colorectal cancer
Spain, 2013	SEOM	SEOM Clinical guidelines for the treatment of advanced colorectal cancer 2013	Colorectal cancer
Spain, 2016	SEOM	SEOM clinical guideline of localized rectal cancer	Rectal cancer
Spain, 2018	SEOM	SEOM clinical guidelines for diagnosis and treatment of metastatic colorectal cancer	Colorectal cancer
UK, 2014	National Institute for Health and Care Excellence	Colorectal cancer: diagnosis and management	Colorectal cancer
UK, 2016	Scottish Intercollegiate Guidelines Network	Diagnosis and management of colorectal cancer	Colorectal cancer
USA, 2012	American Society of Colon and Rectal Surgeons	Practice Parameters for the Management of Colon Cancer	Colon cancer
USA, 2013	American Society of Colon and Rectal Surgeons	Practice Parameters for the Management of Rectal Cancer (Revised	Rectal cancer
USA, 2013	no organization	Managing toxicities associated with antiangiogenic biologic agents in combination with chemotherapy for metastatic colorectal cancer	Colorectal cancer-metastasis
USA, 2015	American Society of Colon and Rectal Surgeons	Practice Guideline for the Surveillance of Patients After Curative Treatment of Colon and Rectal Cancer	Colorectal cancer
USA, 2015	American Cancer Society	American Cancer Society Colorectal Cancer Survivorship Care Guidelines	Colorectal cancer
USA, 2017	American Society of Colon and Rectal Surgeons	The American Society of Colon and Rectal Surgeons clinical practice guidelines for the treatment of colon cancer	Colon cancer
USA, 2018	American Society of Colon and Rectal Surgeons	The American Society of Colon and Rectal Surgeons Clinical Practice Guidelines for Anal Squamous Cell Cancers	Anal cancer
USA, 2018	ASCO	Treatment of patients with early stage colorectal cancer: ASCO resource-stratified guideline	Colorectal cancer
USA, 2018	NCCN	Anal Carcinoma, Version 1.2019	Anal cancer
USA, 2018	NCCN	Colon cancer (MENA edition) v 4.2018	Colon cancer
USA, 2019	ASCO	Duration of oxaliplatin-containing adjuvant therapy for stage III colon cancer: ASCO clinical practice guideline	Colon cancer
USA, 2019	NCCN	Rectal cancer, version 2.2019	Rectal cancer
USA, 2019	NCCN	Colon cancer, version 2.2019	Colon cancer

*ASCO*, American Society of Clinical Oncology; *ESMO*, European Society for Medical Oncology; *ESSO*, European Society of Surgical Oncology; *ESTRO*, European Society of Radiotherapy and Oncology; *EAES*, European Association for endoscopic surgery; *SFCD*, French Society of Gastrointestinal surgery; *ACHBT*, Association of hepatobiliary surgery and liver transplantation; *HESMO*, Hellenic Society of Medical Oncology; *JSMO*, Japanese Society of Medical Oncology; *NHMRC*, National Health and Medical Research Council; *NCCN*, National Comprehensive Cancer Network; *SEOM*, Sociedad Espanola de Oncologia Medica; *SNFGE*, Societe Nationale Francaise de Gastroenterologie; *FFCD*, Federation Francophone de Cancerologie Digestive; *GERCOR*, Groupe Cooperateur multidisciplinaire en Oncologie; *UNICANCER*, Federation Nationale des Centres de Lutte Contre le Cancer; *SFCD*, Societe Francaise de Chirurgie Digestive; *SFED*, Societe Francaise d’Endoscopie Digestive; *SFRO*, Societe Francaise de Radiotherapie Oncologique; *AWMF*, Association of the Scientific Medical Societies in Germany; *DKH*, German Cancer Aid; *DKG*, German Cancer Society
